# Clinicopathological and prognostic value of SIRT6 in patients with solid tumors: a meta-analysis and TCGA data review

**DOI:** 10.1186/s12935-022-02511-3

**Published:** 2022-02-16

**Authors:** Xiaojing Wu, Shuyuan Wang, Xuanzhu Zhao, Sizhen Lai, Zhen Yuan, Yixiang Zhan, Kemin Ni, Zhaoce Liu, Lina Liu, Ran Xin, Xingyu Zhou, Xin Yin, Xinyu Liu, Xipeng Zhang, Wei Cui, Chunze Zhang

**Affiliations:** 1 Department of Colorectal Surgery, Tianjin Union Medical Center, Tianjin, 300121 China; 2 School of Medicine, Nankai University, Tianjin, 300071 China; 3 School of Integrative Medicine, Tianjin University of Traditional Chinese Medicine, Tianjin, 301617 China; 4 School of Mathematical Sciences and LPMC, Nankai University, Tianjin, 300071 China; 5 Tianjin Institute of Coloproctology, Tianjin, 300121 China; 6 Tianjin Medical University, Tianjin, 300041 China

**Keywords:** SIRT6, Solid tumors, Prognosis, Meta-analysis

## Abstract

**Purposes:**

In addition to its role in cellular progression and cancer, SIRT6, a member of nicotinamide adenine dinucleotide (NAD^+^)-dependent class III deacylase sirtuin family, serves a variety of roles in the body's immune system. In this study, we sought to determine the relationship between the expression of SIRT6 and the clinicopathological outcomes of patients with solid tumours by conducting a meta-analysis of the available data.

**Methods:**

The databases PubMed and ISI Web of Science were searched for relevant literature, and the results were presented here. Using Stata16.0, a meta-analysis was conducted to determine the impact of SIRT6 on clinicopathological characteristics and prognosis in malignancy patients. The results were published in the journal Cancer Research. The dataset from the Cancer Genome Atlas (TCGA) was used to investigate the prognostic significance of SIRT6 in various types of tumors.

**Results:**

The inclusion and exclusion criteria were met by 15 studies. In patients with solid tumours, reduced SIRT6 expression was found to be related with improved overall survival (OS) (HR = 0.66, 95% CI = 0.45–0.97, P < 0.001) as well as improved disease-free survival (DFS) (HR = 0.48, 95% CI = 0.26–0.91, P < 0.001). Low SIRT6 expression was found to be associated with a better OS in breast cancer (HR = 0.49, 95% CI = 0.27–0.89, P = 0.179), but was found to be associated with a worse OS in gastrointestinal cancer (gastric cancer and colon cancer) (HR = 1.83, 95% CI = 1.20–2.79, P = 0.939) after subgroup analysis. In terms of clinicopathological characteristics, SIRT6 expression was found to be linked with distant metastasis (OR = 2.98, 95% CI = 1.59–5.57, P = 0.694). When the data from the TCGA dataset was compared to normal tissue, it was discovered that SIRT6 expression was significantly different in 11 different types of cancers. Meanwhile, reduced SIRT6 expression was shown to be associated with improved OS (P < 0.05), which was consistent with the findings of the meta-analysis. Aside from that, the expression of SIRT6 was found to be associated with both gender and clinical stage.

**Conclusion:**

The overall data of the present meta-analysis indicated that low expression of SIRT6 may predict a favorable survival for patients with solid tumors.

**Supplementary Information:**

The online version contains supplementary material available at 10.1186/s12935-022-02511-3.

## Introduction

Sirtuins, a conserved protein family, are identified to have the deacylase and/or mono-ADP-ribosyltransferase activities [1]. Sirtuins have been implicated in a variety of physiological processes, including transcription, DNA repair, tumorigenesis, metabolism, stress responses, apoptosis, fat mobilization, and aging [2]. Among seven identified members (SIRT1-7) of these histone deacetylases, sirtuin 6 (SIRT6) has been shown to be involved in cellular pathways and to play a critical role in regulating ageing and sugar metabolism, both of which are associated with the occurrence and development of tumours and are thus significantly associated with cancer patient prognosis [3, 4]. Against this background, SIRT6 is considered to be a regulator in the progression of cancer and thus affect the survival rate of cancer patients.

SIRT6 expression levels vary significantly between tumour types. Researches reveal that SIRT6 is overexpressed in osteosarcoma [5], papillary thyroid cancer (PTC) [6], prostate cancer [7], conversely reduced in renal cell carcinoma (RCC) [8], pancreatic ductal adenocarcinoma (PDAC) [9], colon cancer (CRC) [10], non-small cell lung cancer (NSCLC) [11]. On the other hand, SIRT6 is thought to play a role in or suppress the progression of several types of cancer. For example, immunohistochemistry revealed that SIRT6 expression was considerably lower in tumour tissues than in normal tissues in RCC patients, implying that SIRT6 worked as a tumour suppressor [8]. Loss of SIRT6 expression in human PDAC defined a subset of patients with a worse prognosis [9]. Moreover, Lower SIRT6 levels were demonstrated in colon cancer and were associated with shorter survival than those of patients with higher SIRT6 expression [10].

In comparison, patients with osteosarcoma who express a high level of SIRT6 exhibit malignant clinical features and have a worse survival rate, with in vitro experiments indicating that SIRT6 overexpression aided MG63 cell motility and invasion [5]. In NSCLC cell lines and tumor tissues, SIRT6 is proved to be upregulated, and statistical analyses showed that high SIRT6-expressing NSCLC patients had a lower cumulative survival rate as compared with low SIRT6-expression patients [11]. SIRT6 upregulation was associated with poor recurrence-free survival (RFS) in PTC patients, given that patients with the higher expression of SIRT6 had the worse RFS and those who possessed lower expression of the gene had the better RFS [6]. Besides, high SIRT6 expression was associated with poor OS of gastric cancer [12].

As a result, while the molecular pathways behind SIRT6 have been explored, the relationship between SIRT6 expression and the prognosis of patients with solid malignancies remains contentious. The goal of this study is to further elucidate the role of SIRT6 in mammalian solid tumours by meta-analysis and the TCGA dataset, which may aid in the detection and treatment of certain cancers.

## Materials and methods

### Study research

We conducted an electronic literature search of all publications in the PubMed and ISI Web of Science databases to determine whether there is a correlation between SIRT6 expression and survival in solid tumours. The research was terminated on August 15, 2021, with no lower date limit. Sirtuin 6, SIRT6, cancer, tumour, prognosis, prognostic, and survival were all included in the search terms in all possible combinations. Searches were limited to human studies and those published in English.

### Inclusion and exclusion criteria

The included studies had to meet the following criteria: (1) to be published in its entirety in the English language; (2) to make a pathological diagnosis of cancer; (3) to describe the pathological diagnosis of various tumour types or clinicopathological features; (4) to measure SIRT6 expression in patients with any type of tumour via immunohistochemistry; (5) to describe associations between SIRT6 expression and OS and DFS; and (6) to report or calculate HRs and 95% CIs based on the information in the pamphlet.

The following criteria were used to exclude studies from this meta-analysis: (1) Reviews, letters, comments, repetitive research, case reports, or personal communications; (2) non-English language articles; (3) articles that overlap or contain duplicate data; (4) articles that contain only animal experiments; and (5) studies that do not include survival curves or data on survival.

### Data extraction

Two investigators (Shuyuan Wang and Zhen Yuan) extracted all data independently based on the inclusion and exclusion criteria, and all items were finally agreed upon. The following characteristics were extracted for each eligible study: the first author’s name, the publication year, the region, the type of cancer, the number of patients, the duration of follow-up, the detection methods, the survival data (including OS and DFS), and clinicopathological parameters such as gender, tumour differentiation, T status, lymph node metastasis, distant metastasis, and TNM stage.

### Statistical analysis

Stata 16.0 (Stata Corporation, College Station, TX, USA) was used to conduct this meta-analysis. The association between SIRT6 expression and survival outcome was evaluated using pooled HR estimates with 95% CIs, while the association with clinical parameters such as gender, tumour differentiation, lymph node metastasis, distant metastasis, and clinical stage was evaluated using OR estimates with 95% CIs. Further, the Cochrane’s Q test and I^2^ statistical test were used to analyze the heterogeneity between studies, the fixed effects model (Mantel–Haenszel method) was conducted when heterogeneity was negligible (I^2^ < 50%), and a random effects model (DerSimonian-Laird method) was used when heterogeneity was significant (I^2^ > 50%). Begg’s funnel plot was conducted to identify publication bias. All the P values were used for a two-sided test with significance at P < 0.05. The HRs and 95% CIs were extracted from articles that only reported Kaplan–Meier curves using Software Engauge Digitizer (version 10.8). Tierney et al. provided the method and EXCEL programmed for calculating the data [13].

### Extraction and analysis of TCGA dataset

The Cancer Genome Atlas (TCGA) dataset (https://www.cancer.gov/about-nci/organization/ccg/research/structural-genomics/tcga) and UCSC Xena project (https://xena.ucsc.edu/) were used to extract the data in malignant tumors and normal tissues, including the expression of SIRT6, survival data and clinicopathological parameters. The differential expression of SIRT6 between tumours and paired normal tissues was analyzed using Wilcoxon’s signed-rank test. The OS and DFS curves were generated using the Kaplan–Meier method and the logrank test, respectively, using data from the TCGA dataset. To analyse the prognostic effects of SIRT6 in various tumours, the forest plot was mapped using univariate Cox regression. The correlation between SIRT6 expression and clinicopathological parameters were determined using a one-way ANOVA. All data were analyzed and plots were created using the R programming language (version 3.6.1).

## Results

### Literature search and study characteristics

**Fig. 1:**
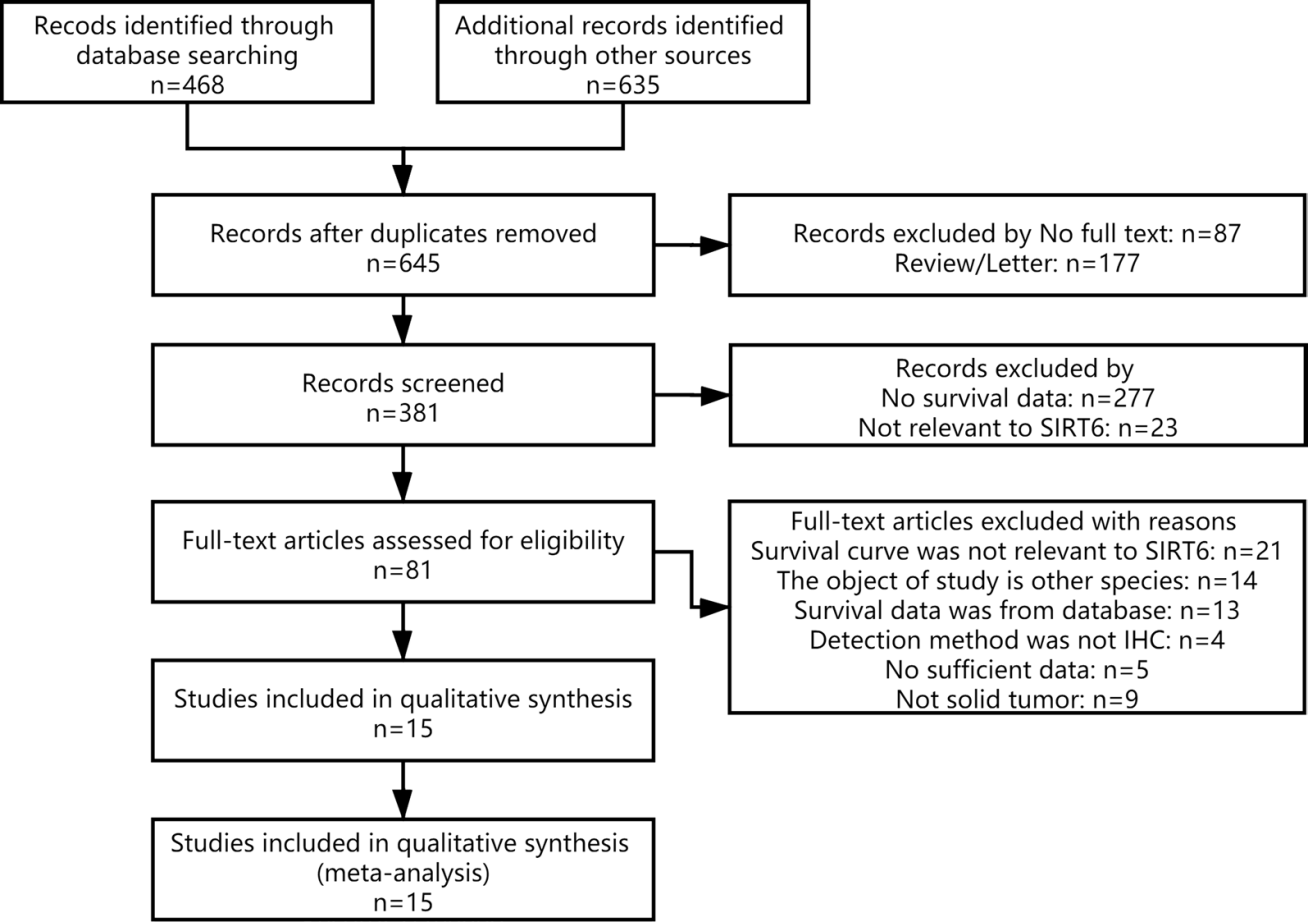
Flow diagram of the selection of eligible studies

A total of 1103 publications were discovered in the PubMed and ISI Web of Science databases. Figure 1 depicts a detailed study selection. 1088 of those were excluded due to duplication of research, lack of complete texts, lack of information about survival, detection method, or studies irrelevant to the current analysis. Finally, 15 publications were included in this study, totaling 1577 patients. Table 1 summaries the major characteristics of the 15 eligible studies.

**Table 1:** Main characteristics of studies exploring the relationship between SIRT6 expression and tumor prognosis

Authors	Year	Region	Cancer type	Stage/ Grade	No. of Patients	Follow-up Time Median (range)	Detection Method	Outcomes	Location	NOS Score
Zhang [14]	2020	Korea	OS	I–IV	37	NR	IHC (CST)	OS, DFS	NR	8
Han [15]	2019	China	HCC	NR	120	60 M	IHC (Bioss)	OS, DFS	NR	7
Bae [16]	2018	Korea	OC	I–IV	104	82 M(1–209)	IHC (CST)	OS, DFS	Nu	8
Tian [10]	2018	China	CRC	I–IV	90	NR	IHC (CST)	OS	Nu	7
Li [17]	2018	China	CRC	I–IV	97	NR	IHC (Abcam)	OS	Nu	7
Zhu [18]	2018	China	NSCLC	I–IV	86	51 M	IHC (Abcam)	OS, DFS	Nu	6
Chen [4]	2017	China	NSCLC	I–III	122	44 M(1–60)	IHC (Abcam)	OS	Cy	7
Zhou [19]	2017	China	GC	I–IV	68	NR	IHC (CST)	OS, DFS	NR	6
Bai [11]	2016	China	NSCLC	I–IV	174	30 M(0–120)	IHC (Abcam)	OS	NR	5
Bae [20]	2016	Korea	BRC	I–IV	142	148.8 M(7.7–198.6)	IHC (Lifespan)	OS, DFS	Nu	7
Kugel [9]	2016	USA	PDAC	NR	120	33 M	IHC (NR)	OS	NR	9
Ran [21]	2016	China	HCC	I–III	53	NR	IHC (Novus)	OS	NR	8
Azuma [22]	2015	Japan	NSCLC	I–IV	98	NR	IHC (Abnova)	OS	Cy	7
Thirumurthi [23]	2014	China	BRC	NR	126	NR	IHC (CST)	OS	NR	8
Khongkow [24]	2013	China	BRC	I–III	118	NR	IHC (CST)	OS	Nu	8

*NR* not report, *M* month, *IHC* immunohistochemistry, *OC* ovarian carcinomas, *CRC* colon cancer, *NSCLC* non-small cell lung cancer, *GC* gastric cancer, *PDAC* pancreatic ductal adenocarcinoma, *HCC* hepatocellular carcinoma, *BRC* breast cancer, *OS* osteosarcoma, *Nu* nucleus, *Cy* cytoplasm

### Decreased SIRT6 expression and overall survival

**Fig. 2:**
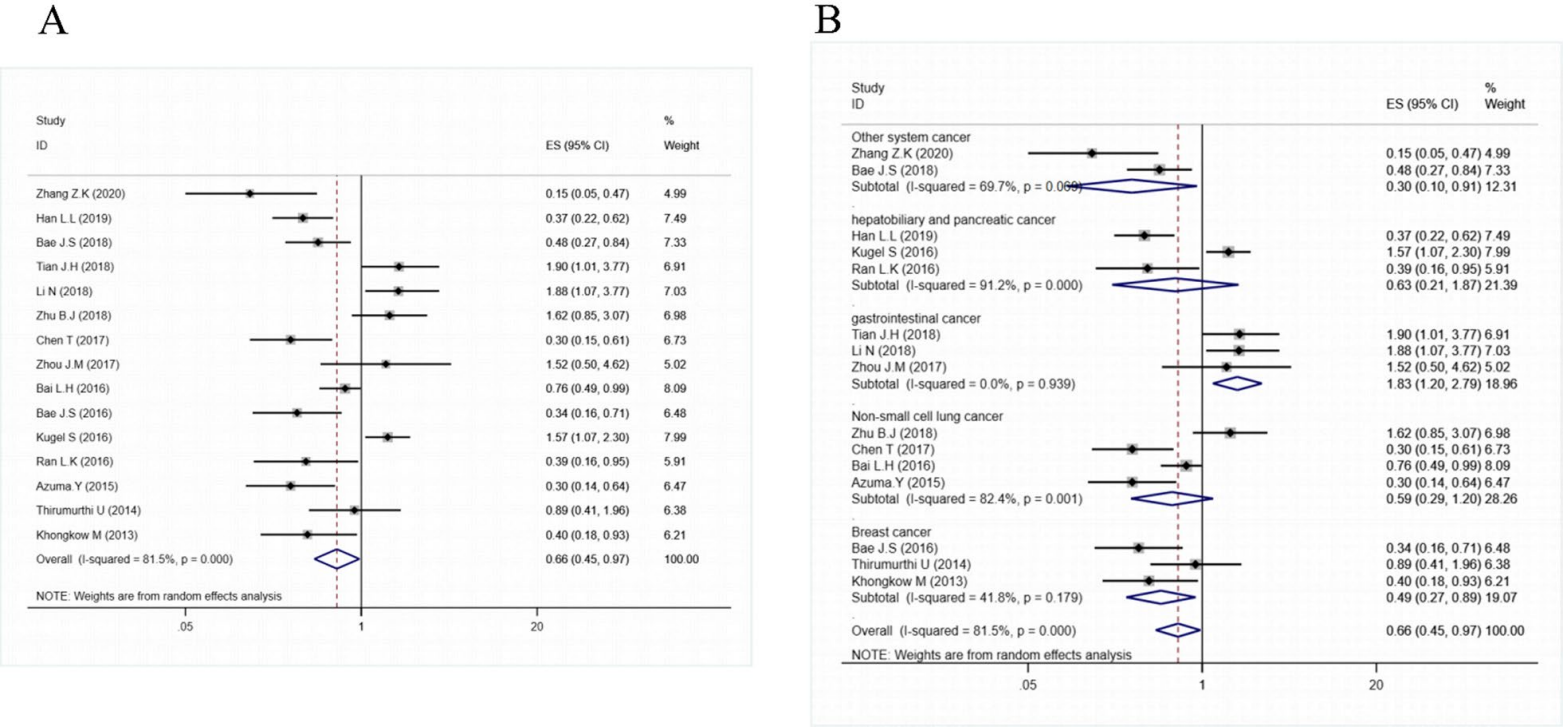
**A** Forest plot of the association between SIRT6 expression and OS; **B** subgroup analysis by tumor type

The association between SIRT6 expression and the prognosis for OS was estimated; the pooled HR and 95% CIs are shown in Fig. 2A. The findings demonstrated that low SIRT6 expression was associated with a longer overall survival in patients with solid cancer (HR = 0.66, 95% CI = 0.45–0.97, P < 0.001). As shown in Fig. 2B, we conducted a subgroup analysis by cancer type, which revealed that low SIRT6 expression was significantly associated with better overall survival in breast cancer (HR = 0.49, 95% CI = 0.27–0.89, P = 0.179) and other system cancer (OS and OC) (HR = 0.30, 95% CI = 0.10–0.91, P = 0.069), but not in non-small cell lung cancer, hepatobiliary and pancreatic cancer. However, Furthermore, decreased SIRT6 expression was found to be associated with a shorter overall survival (OS) in gastrointestinal tumours (GC and CRC) (HR = 1.83, 95% CI = 1.20–2.79, P = 0.939).

### Decreased SIRT6 expression and disease-free survival

**Fig. 3:**
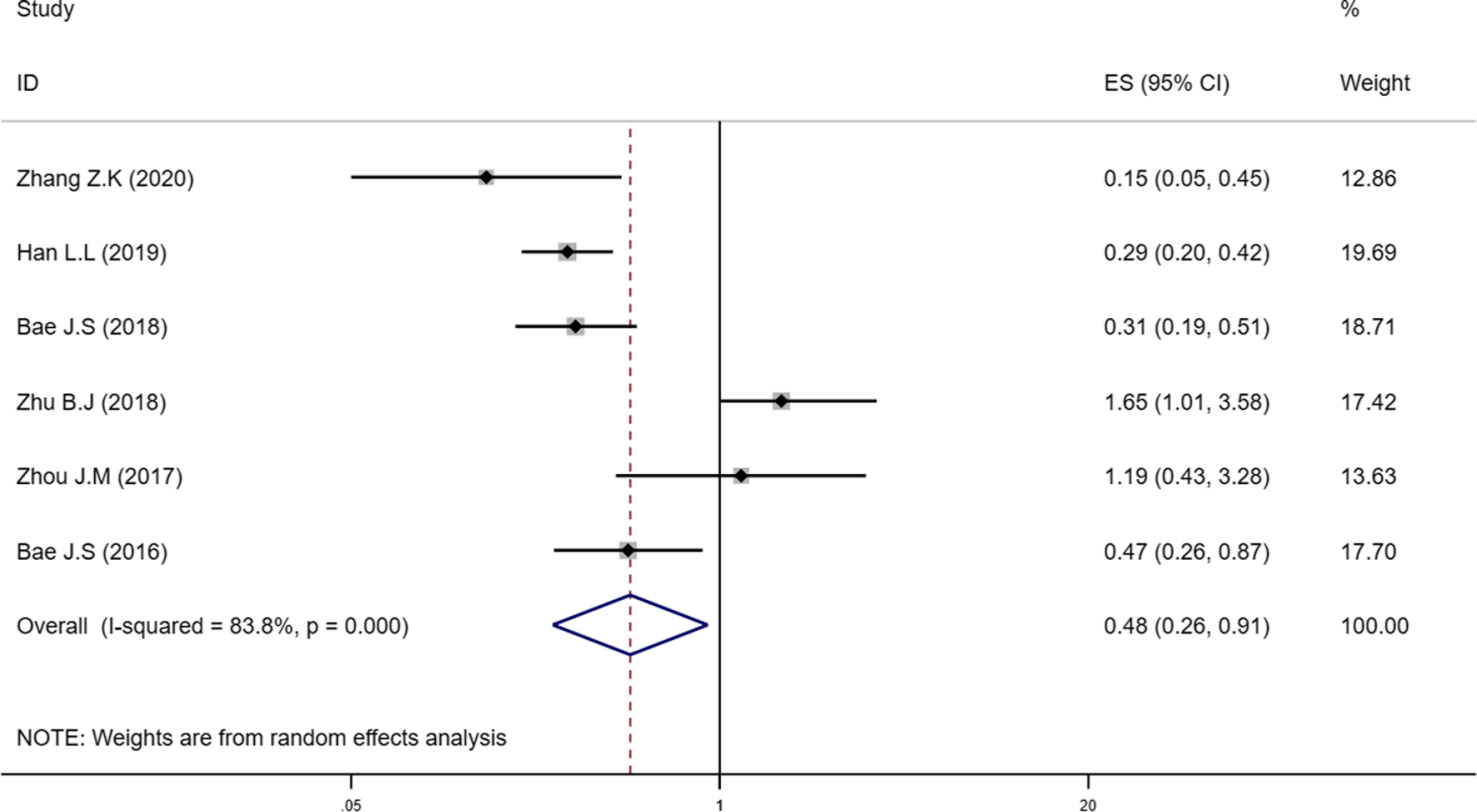
Forest plot describing the association between low-expressed SIRT6 and DFS

The association between SIRT6 expression and prognosis for DFS has been estimated; the pooled HRs and 95% CIs are shown in Fig. 3. In a multivariate analysis of patients with solid tumours, a significant correlation between attenuated SIRT6 expression and DFS (HR = 0.48, 95% CI = 0.26–0.91) was observed in the random effects model with a significant heterogeneity (I^2^ = 83.8%, P < 0.001).

### Correlations between Low SIRT6 Expression and Clinicopathological parameters

12 eligible articles were used to collect the clinical and pathological parameters, owing to 3 articles [9, 23, 24] did not provide related information, the specific parameters were presented in Additional file 4: Table S1. As shown in Table 2, correlations between attenuated-expressed SIRT6 and clinicopathological characteristics of patients with solid tumours were discovered using pooled results of the correlations. Reduced SIRT6 expression was found to be associated with distant metastasis (OR = 2.98, 95% CI = 1.59–5.57, P = 0.694). No significant correlations between low expressed SIRT6 and gender (OR = 0.92, 95% CI = 0.63–1.33, P = 0.226), tumor differentiation (OR = 1.35, 95% CI = 0.68–2.67, P = 0.000), T status (OR = 1.24, 95% CI = 0.70–2.18, P = 0.018), lymph node metastasis (OR = 0.86, 95% CI = 0.55–1.36, P = 0.010) and TNM stage (OR = 0.69, 95% CI = 0.35–1.34, P = 0.001) were observed when the P-value was controlled to be < 0.05 (Additional file 1: Fig. S1).

**Table 2:** Meta-analysis results of the associations of decreased SIRT6 expression with clinicopathological parameters

Clinicopathological parameter	Study Number	Overall OR (95% CI)	I^2^ ( P-value)
Gender (male vs female)	9	0.92 (0.63–1.33)	24.5%, 0.226
Tumor differentiation (poor/moderate vs well)	8	1.35 (0.68–2.67)	77.8%, < 0.001
T status (T3-4 vs T1-2)	9	1.24 (0.70–2.18)	56.7%, 0.018
Lymph node metastasis (yes vs no)	8	0.86 (0.55–1.36)	58.7%, 0.010
Distant metastasis (yes vs no)	3	2.98 (1.59–5.57)	0.00%, 0.694
TNM stage (III-IV vs I-II)	7	0.69 (0.35–1.34)	70.6%, 0.001

If 95% CI value contain 1.0, it means that the 95% CI value intersect the ineffective line and the OR value was no statistical significance

### Assessment of heterogeneity and sensitivity analysis

Because of the high heterogeneity of the meta-analysis with OS, subgroup analyses were performed according to the SIRT6 location (nucleus or cytoplasm), patients' country (China or other), case number (≥ 100 or not), NOS score (> 7 or not), and publication year (≥ 2018 or ≤ 2017), among other variables. Low expression of SIRT6 in the cytoplasm was associated with improved OS; however, the expression of SIRT6 in the nucleus or in a location that was not reported was not associated with OS. Apart from that, Table 3 shows the pooled HRs and heterogeneities according to all of these variables. All of these subgroup analyses revealed that the I2 value was not significantly lower when the P value was less than 0.05 in any of the groups. Therefore, the subgroup analysis failed to identify the source of extreme heterogeneity (Additional file 2: Fig. S2, Additional file 3: Fig. S3).

**Table 3:** Results of subgroup analysis exploring source of heterogeneity with OS and DFS

Subgroups	OS		DFS	
	HR (95% CI)	I^2^ (P-value)	HR (95% CI)	I^2^ (P-value)
Location				
Nu	0.86 (0.45–1.66)	82.5% (P < 0.001)	0.62 (0.26–0.91)	88.2% (P < 0.001)
Cy	0.30 (0.18–0.50)	0.0% (P = 1.000)	–	–
NR	0.65 (0.38–1.13)	81.8% (P < 0.001)	0.37 (0.14–0.97)	76.5% (P = 0.014)
Region				
China	0.79 (0.51–1.23)	78.0% (P < 0.001)	0.80 (0.22–2.88)	91.9% (P < 0.001)
Other	0.44 (0.19–1.03)	88.4% (P < 0.001)	0.32 (0.19–0.54)	41.8% (P = 0.180)
Patients number				
≥ 100	0.56 (0.36–0.88)	80.7% (P < 0.001)	0.48 (0.26–0.91)	0.0% (P = 0.384)
< 100	0.80 (0.39–1.65)	82.9% (P < 0.001)	0.70 (0.18–2.78)	85.7% (P = 0.001)
NOS score				
> 7	0.54 (0.27–1.07)	82.8% (P < 0.001)	0.26 (0.14–0.48)	28.3% (P = 0.237)
≤ 7	0.74 (0.45–1.23)	82.8% (P < 0.001)	0.48 (0.26–0.91)	87.9% (P < 0.001)
Publication year				
≥ 2018	0.77 (0.37–1.60)	87.1% (P < 0.001)	0.40 (0.17–0.94)	88.5% (P < 0.001)
≤ 2017	0.59 (0.37–0.94)	78.3% (P < 0.001)	0.48 (0.26–0.91)	83.8% (P < 0.001)

**Fig. 4:**
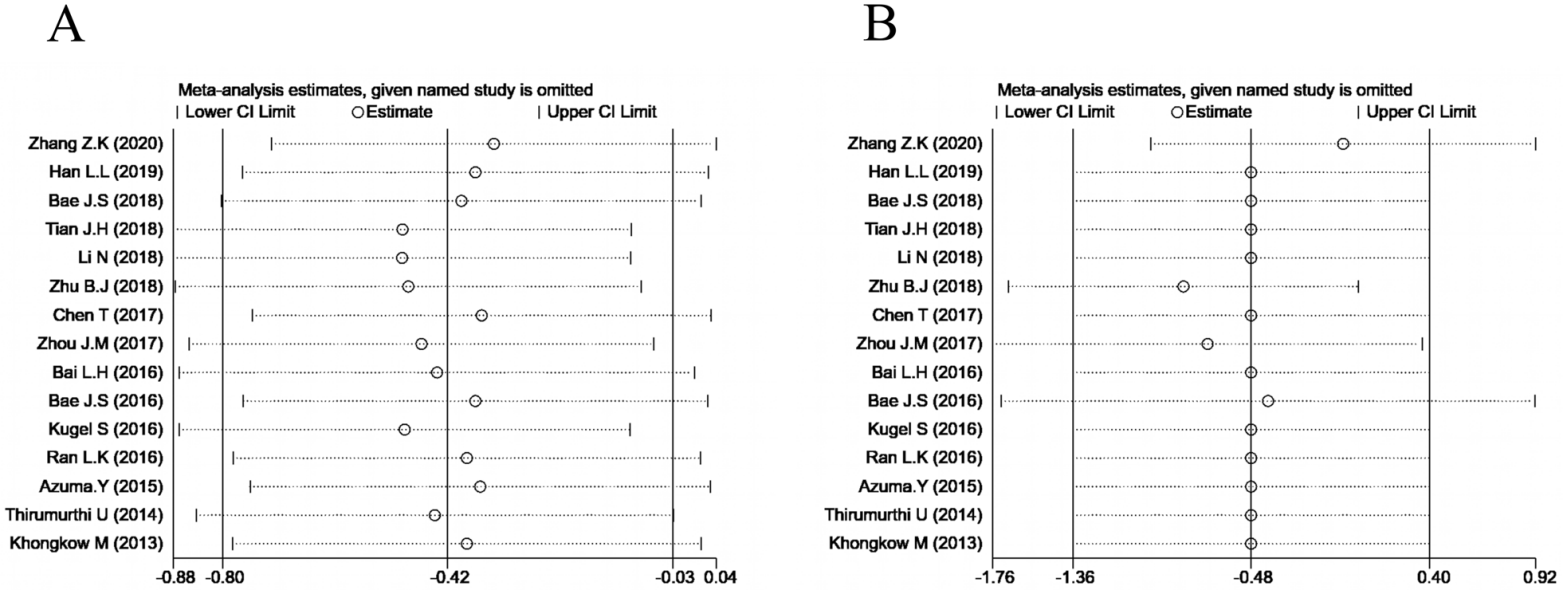
Sensitivity analysis of each study. **A** Sensitivity analysis for OS; **B** Sensitivity analysis for DFS

Additional to this, a sensitivity analysis was carried out by systematically excluding each study from the aggregated survival meta-analyses, in order to assess the influence of each individual study on the pooled HR of OS (Fig. 4). The results revealed that the pooled estimates of the effect of low-expressed SIRT6 on the OS and DFS of patients with solid tumours did not differ significantly when individual studies were excluded, implying that the findings of this meta-analysis were stable.

### Publication bias

**Fig. 5:**
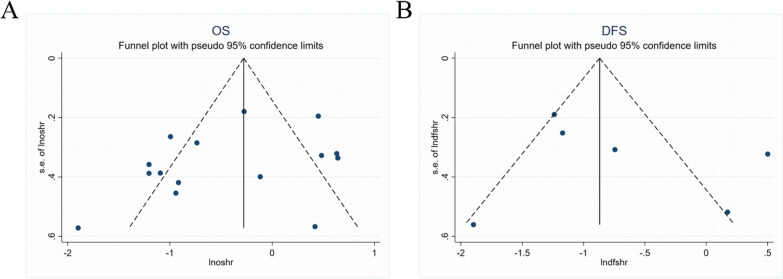
Funnel plot for publication bias. **A** Funnel plot for OS; **B** Funnel plot for DFS

**Fig. 6:**
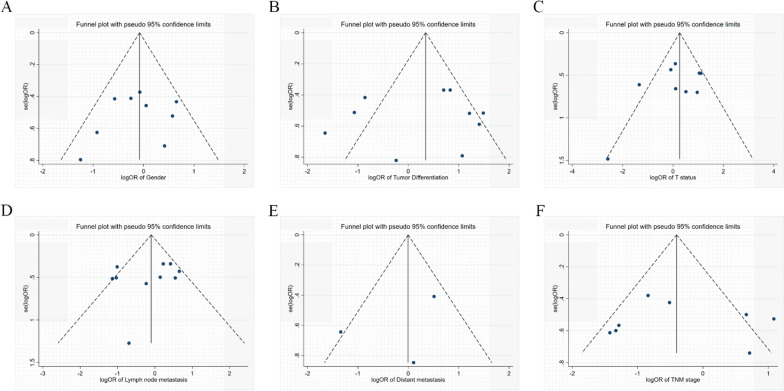
Funnel plot for publication bias regarding clinicopathological features. **A** Gender; **B** Tumor differentiation; **C** T status; **D** Lymph node metastasis; **E** Distant metastasis; **F** TNM stage

The shapes of the funnel plots for OS, DFS and clinicopathological features of patients were almost symmetrical, indicated that there was no statistically significant difference, therefore no significant publication bias (Figs. 5, 6). Thus, in these incorporated papers, it was found that there was no evidence of significant publication bias.

### Validation of the results in TCGA dataset

For further investigation, the OS and DFS data of patients with expression of SIRT6 in different cancers were extracted from the TCGA dataset, including 17 types of tumors, Adrenocortical carcinoma (ACC), Bladder Urothelial Carcinoma (BLCA), Breast invasive carcinoma (BRCA), Cervical squamous cell carcinoma and endocervical adenocarcinoma (CESC), Cholangiocarcinoma (CHOL), Colon adenocarcinoma (COAD), Esophageal carcinoma (ESCA), Glioblastoma multiforme (GBM), Head and Neck squamous cell carcinoma (HNSC), Kidney Chromophobe (KIRC), Kidney Chromophobe (KICH), Testicular Germ Cell Tumors (TGCT), Thymoma (THYM), Thyroid carcinoma (THCA), Uterine Corpus Endometrial Carcinoma (UCEC), Uterine Carcinosarcoma (UCS) and Uveal Melanoma (UVM).

**Fig. 7:**
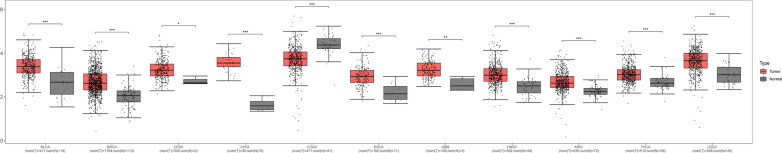
Expression of SIRT6 was significantly different in 11 malignant tumor tissues (red) and paired normal tissues (gray). Bladder Urothelial Carcinoma (BLCA); Breast invasive carcinoma (BRCA); Cervical squamous cell carcinoma and endocervical adenocarcinoma (CESC); Cholangiocarcinoma (CHOL); Colon adenocarcinoma (COAD); Esophageal carcinoma (ESCA); glioblastoma multiforme (GBM); Head and Neck squamous cell carcinoma (HNSC); Kidney Chromophobe (KIRC); Thyroid carcinoma (THCA) and Uterine Corpus Endometrial Carcinoma (UCEC)

**Fig. 8:**
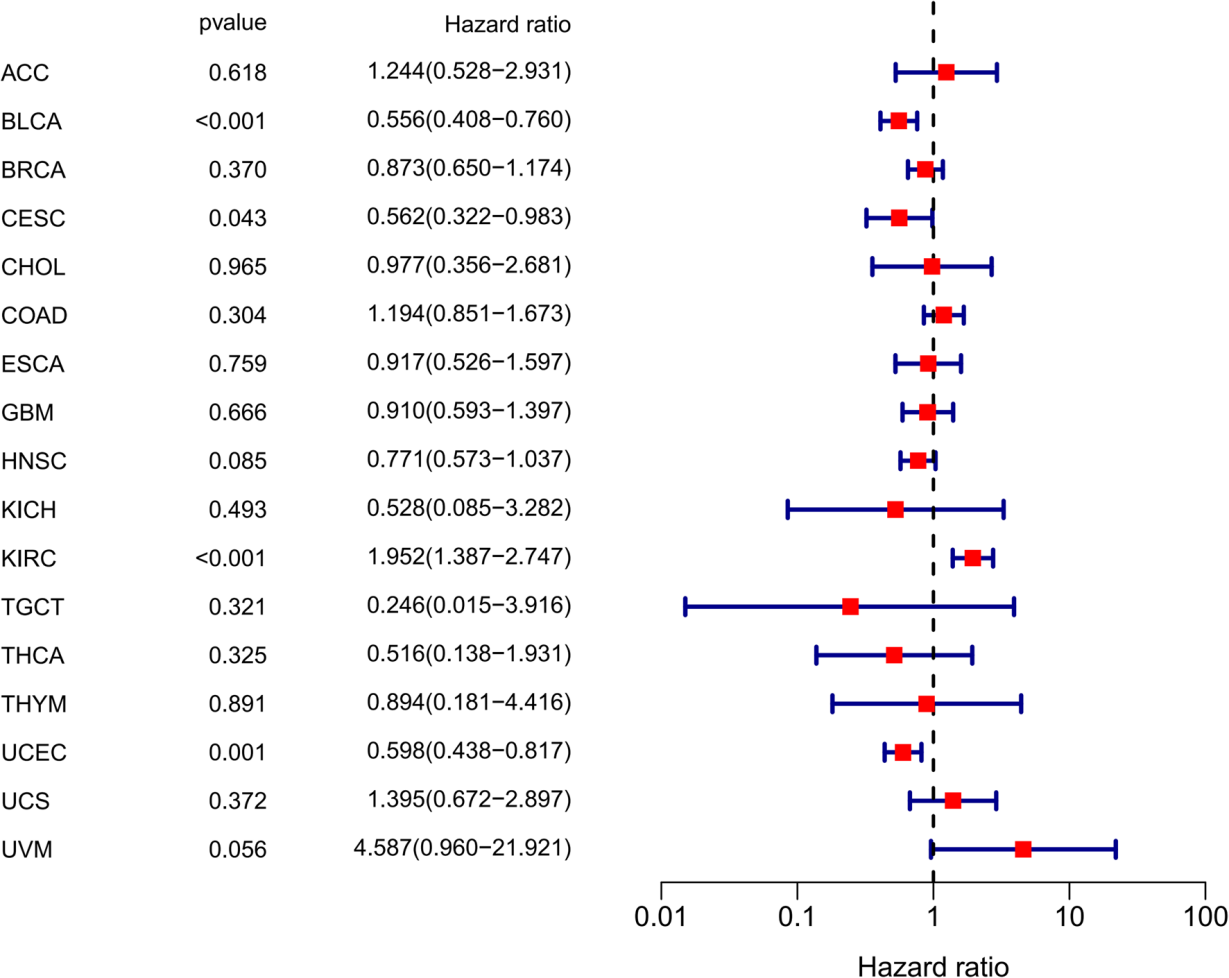
Forest plot for prognosis of SIRT6 in tumors analyzed by univariate Cox regression in TCGA. Adrenocortical carcinoma (ACC), Kidney Chromophobe (KICH), Testicular Germ Cell Tumors (TGCT), Thymoma (THYM), Uterine Carcinosarcoma (UCS) and Uveal Melanoma (UVM)

As illustrated in Fig. 7, SIRT6 expression was significantly different in 11 different types of malignant tumours compared to normal tissues (P < 0.05). SIRT6 was found to be a high-risk gene in KIRC but a low-risk gene in BLCA and UCEC using univariate Cox regression (Fig. 8).

**Fig. 9:**
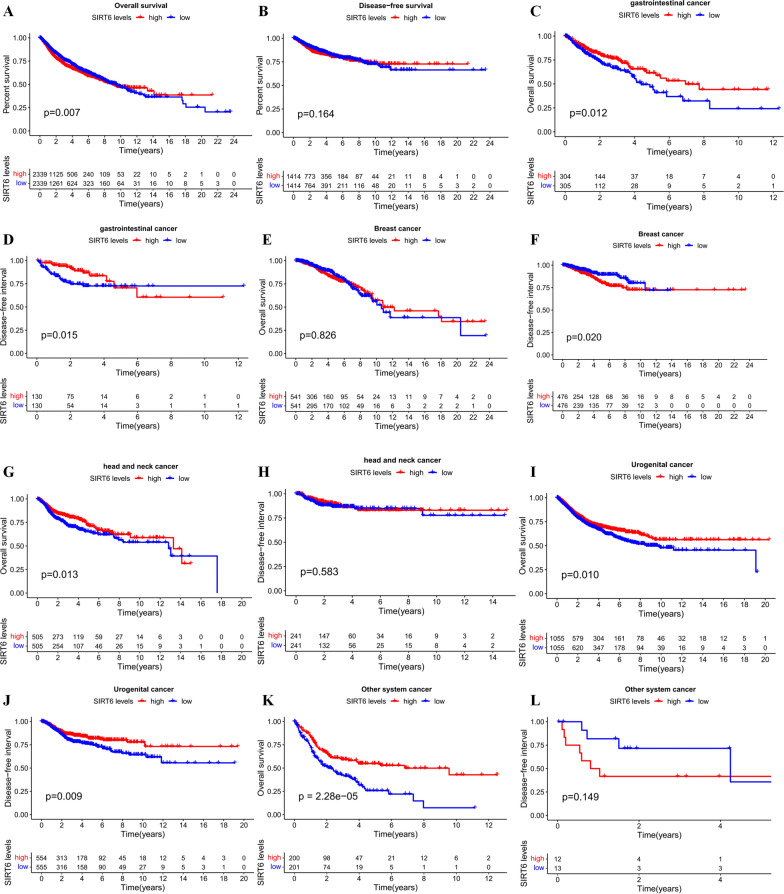
Kaplan–Meier survival curves of different tumor types of patients in TCGA dataset. **A** Overall survival plot of SIRT6 in all 17 types of tumors (n = 4678, logrank P < 0.05); **B** Disease-free survival plot of SIRT6 in all 17 types of tumors (n = 2828, logrank P = 0.164); **C** Overall survival plot of SIRT6 in gastrointestinal cancer (n = 609, logrank P < 0.05); **D** Disease-free survival plot of SIRT6 in gastrointestinal cancer (n = 260, logrank P < 0.05); **E** Overall survival plot of SIRT6 in breast cancer (n = 1082, logrank P = 0.826); **F** Disease-free survival plot of SIRT6 in breast cancer (n = 952, logrank P < 0.05); **G** Overall survival plot of SIRT6 in head and neck cancer (n = 1010, logrank P < 0.05); **H** Disease-free survival plot of SIRT6 in head and neck cancer (n = 482, logrank P = 0.583); **I** Overall survival plot of SIRT6 in urogenital cancer (n = 2130, logrank P < 0.05); **J** Disease-free survival plot of SIRT6 in urogenital cancer (n = 1109, logrank P < 0.05); **K** Overall survival plot of SIRT6 in other system cancer (n = 401, logrank P < 0.05); **L** Disease-free survival plot of SIRT6 in other system cancer (n = 25, logrank P = 0.149)

By merging SIRT6 expression data and the survival data of all types of tumors included, we found that the low expression of SIRT6 denoted a better OS (P < 0.05) (Fig. 9A), but was not significantly associated with worse DFS (Fig. 9B), which consistent with the meta-analysis. We also divided the data into five subgroups, including gastrointestinal cancer (including ESCA and COAD), head and neck cancer system (including HNSC and THCA), urogenital cancer (including BLCA, KICH, KIRC, ACC, TGCT, UCEC, CESC and USC), breast cancer and other system cancer (including GBM, THYM, UVM and CHOL).

The results revealed that decreased SIRT6 was significantly associated with unfavorable OS (P < 0.05) (Fig. 9C) and DFS (P < 0.05) (Fig. 9D) in gastrointestinal tumors (P < 0.05), which validated the results of meta-analysis. There was no significantly correlation between SIRT6 expression and OS (Fig. 9E) observed in breast cancer. However, downregulated SIRT6 favored better DFS in patients with breast cancer (P < 0.05) (Fig. 9F). Further, low SIRT6 expression predicted worse OS in head and neck cancer (P < 0.05) (Fig. 9G), urogenital cancer (P < 0.05) (Fig. 9I) and other system cancer (P < 0.05) (Fig. 9K).

**Fig. 10:**
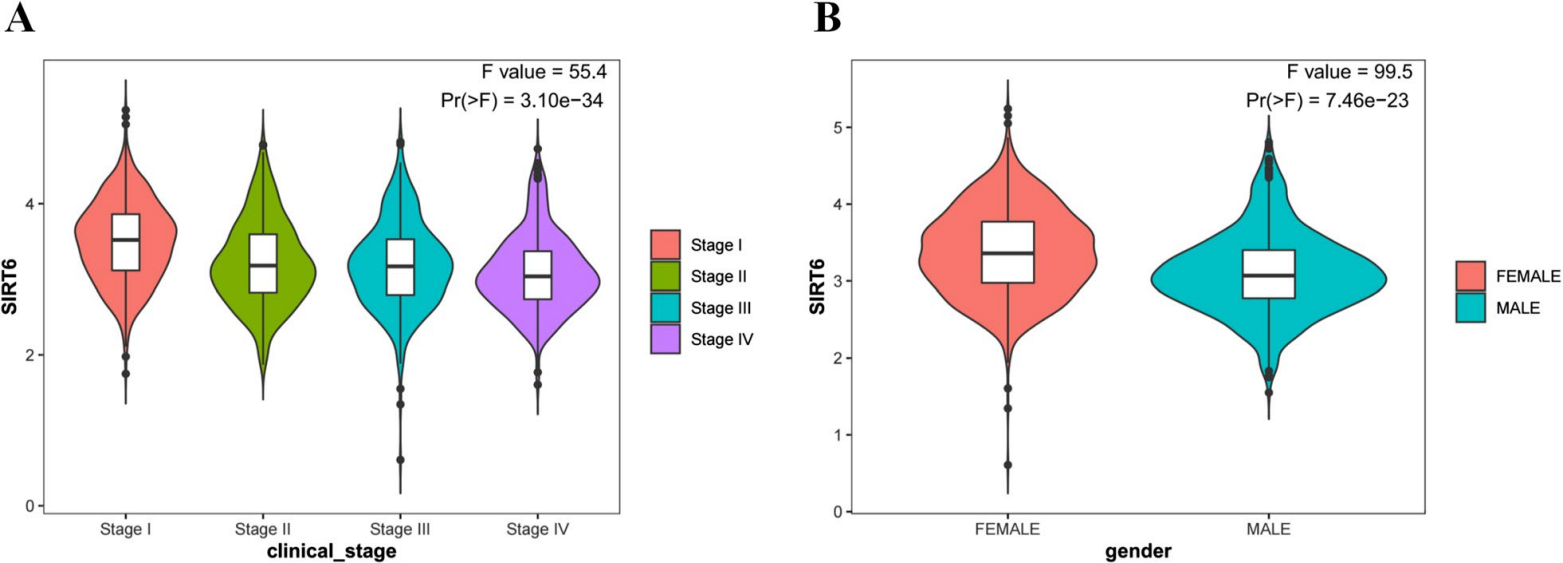
Violin plot demonstrated that expression of SIRT6 was significantly associated with clinical stage (P < 0.001) and gender (P < 0.001). **A** Clinical stage; **B** Gender

We also evaluated the correlation between SIRT6 expression and clinicopathological parameters. As shown in the violin plot (Fig. 10), the expression of SIRT6 was significantly associated with clinical stage (P < 0.05) and gender (P < 0.05), but not associated with distant metastasis.

## Discussion

SIRT6, a key member of the long-lived protein family, has been shown to regulate a variety of physiological processes and is intimately involved in tumour formation and progression. The current meta-analysis, which included 15 studies and 1577 patients, was the first to summarise all previously published research on the effect of SIRT6 expression on human tumour prognosis. It established a significant association between SIRT6 expression and a decline in cancer patients’ OS (HR = 0.66, 95% CI = 0.45–0.97, P < 0.001) and DFS (HR = 0.48, 95% CI = 0.26–0.91). Low SIRT6 expression was associated with a better OS in breast cancer (HR = 0.49, 95% CI = 0.27–0.89, P = 0.179), but was associated with a worse OS in gastrointestinal tumours (HR = 0.30, 95% CI = 0.10–0.91, P = 0.069).

Additionally, multivariate analysis revealed a correlation between low cytoplasmic SIRT6 expression and improved OS (HR = 0.30, 95% CI = 0.18–0.50, P = 1.000). SIRT6 deficiency was associated with distant metastasis (OR = 2.98, 95% CI = 1.59–5.57, P = 0.694). However, no obvious correlations between decreased SIRT6 expression and other clinicopathological characteristics were observed.

Additionally, the TCGA dataset was used to assess SIRT6's prognostic value in various tumour types. The TCGA dataset revealed that decreased SIRT6 was associated with a better overall survival in all 17 types of tumours, but with a worse overall survival in gastrointestinal cancer, which was consistent with meta-analysis results. However, low SIRT6 expression has been associated with a poorer OS in head and neck cancer, urogenital cancer, and other system cancers.

The biological function of SIRT6 may help to explain the contradictory findings. The current study demonstrated that SIRT6 acts as a double-edged sword during the development of solid tumours, suppressing or promoting tumour growth, depending on the type of tumour [25]. However, in the same tumor type, SIRT6 may also play dual roles in tumor progression by activating different signaling pathways, such as breast cancer [26, 27] and HCC [21]. In the context of cancer inhibition, the tumor suppression function of SIRT6 may achieved by regulating DNA repair, genomic stability, metabolic homeostasis and apoptosis [28], of which the main mechanism was the suppression of aerobic glycolysis (a.k.a. Warburg effect), an common alteration in glucose metabolism in cancer cells [29]. Further, upregulated SIRT6 achieves the function of tumor suppression in HCC through inhibiting phosphorylation of ERK1/2 [30] and reducing the expression of cycling D1 and p-ERK [31]. SIRT6 can not only upregulate the expression of tumor suppressors phosphatase and tensin (PTEN), and phosphatidylinositol-4,5-biphosphate (PIP2), but also can downregulate AKT1, mTOR, cyclin D1, and c-myc to inhibit the progression of colon cancer [10]. The role of SIRT6 in tumour promotion has been extensively studied in recent years. SIRT6 can inhibit Bax activation caused by H3K9 deacetylation, thereby promoting HCC growth [21]. SIRT6 promotes breast cancer by regulating the acetylation and sensitivity to lapatinib of Forkhead box protein O3 (FoxO3) [32].

Both of SIRT6's functions in the same tumour type may be related to autophagy. Autophagy can degrade toxic proteins and dysfunctional organelles in the early stages of cancer, but in the later stages, the sensitivity of cancer cells to pressure may be reduced, which can aid in the progression of the disease and its progression [33]. Meanwhile, SIRT6 induced DNA repair can inhibit the development of tumors in initial phase, whereas promote the growth of tumors in later phase [28].

The results of the TCGA dataset were not entirely consistent with the conclusions of the meta-analysis, which could be attributed to any of the factors listed above. Furthermore, other factors that are not mentioned in the papers, such as the detection method, the detection phase, whether or not p53 is phosphorylated, and other factors, may contribute to the differences between the results.

The original articles included in this study were all prospective, which reduced the likelihood of selection bias and reverse causation to a great extent. A large number of cases had been gathered from various studies, and the total number of participants (1577) was significant, increasing the statistical power of the analysis. The results of the funnel plot and Begg’s analysis did not reveal any evidence of publication bias, indicating that the findings have a high degree of credibility. However, there were some mediocre imitations found in this study that were worth mentioning. The main imitation was the high heterogeneity between overall survival, disease-free survival, and various clinicopathological parameter analyses. Furthermore, subgroup analysis did not yield a clear picture of the source. According to the documents and materials available, we know that while sirtuins can be found in a variety of cellular compartments, SIRT6 is primarily found in the nucleus, where it can bind and deacylate chromatin as well as other substrates, the majority of which are transcription factors (Fig. 1) [34–36]. Throughout the 15 selected studies, there are only 8 articles reported the SIRT6 location, which may be a source of high heterogeneity.

Meanwhile, other possible knockoffs should be taken into consideration as well. First and foremost, we require more trials to analyze; second, we require articles that investigate a greater variety of cancer types; third, some of the survival data was extracted from Kaplan–Meier curves, which may be less reliable than a direct analysis of variance; and fourth, we require more non-English publications to search.

## Conclusion

In conclusion, despite some limitations, our meta-analysis results convincingly demonstrated that decreased SIRT6 expression, as measured by immunohistochemistry, is positively associated with improved overall survival and disease-free survival in patients with solid tumours. Low SIRT6 expression may serve as a potential biomarker for improved survival outcomes in patients with a variety of solid tumours, suggesting that therapeutic approaches that directly target SIRT6 may be promising for the treatment of solid malignancies.

## Supplementary Information

**Additional file 1: Figure S1.** Forest plot for different clinicopathological parameters.

**Additional file 2: Figure S2.** Forest plot for OS of different clinicopathological parameters.

**Additional file 3: Figure S3.** Forest plot for DFS of different clinicopathological parameters.

**Additional file 4: Table S1.** Summarized data of clinical and pathological parameters from the eligible studies.

## Data Availability

The datasets of meta-analysis used in this study are available from the corresponding author upon reasonable request. The data in TCGA analysis are available at https://www.cancer.gov/about-nci/organization/ccg/research/structural-genomics/tcga and https://xena.ucsc.edu/.

## Abbreviations

SIRT6: Sirtuin-6
OS: Overall survival
DFS: Disease-free survival
RFS: Recurrence-free survival
TCGA: The Cancer Genome Atlas
PTC: Papillary thyroid cancer
RCC: Renal cell carcinoma
CI: Confidence interval
I^2^: Inconsistency index
HR: Hazard ratios
NOS: Newcastle Ottawa Scale
IHC: Immunohistochemistry
OC: Ovarian carcinomas
CRC: Colon cancer
NSCLC: Non-small cell lung cancer
GC: Gastric cancer
PDAC: Pancreatic ductal adenocarcinoma
HCC: Hepatocellular carcinoma
BRC: Breast cancer
OS: Osteosarcoma
Nu: Nucleus
Cy: Cytoplasm
