# Supplementary material for: Clinicopathological and prognostic value of SIRT6 in patients with solid tumors: a meta-analysis and TCGA data review
Source: Cancer Cell Int. 2022 Feb 16;22:84. doi: 10.1186/s12935-022-02511-3 (PMC8848894; doi:10.1186/s12935-022-02511-3)
Supplement: Supplementary file 1 — Additional file 1: Figure S1. Forest plot for different clinicopathological parameters. [file 12935_2022_2511_MOESM1_ESM.pdf]

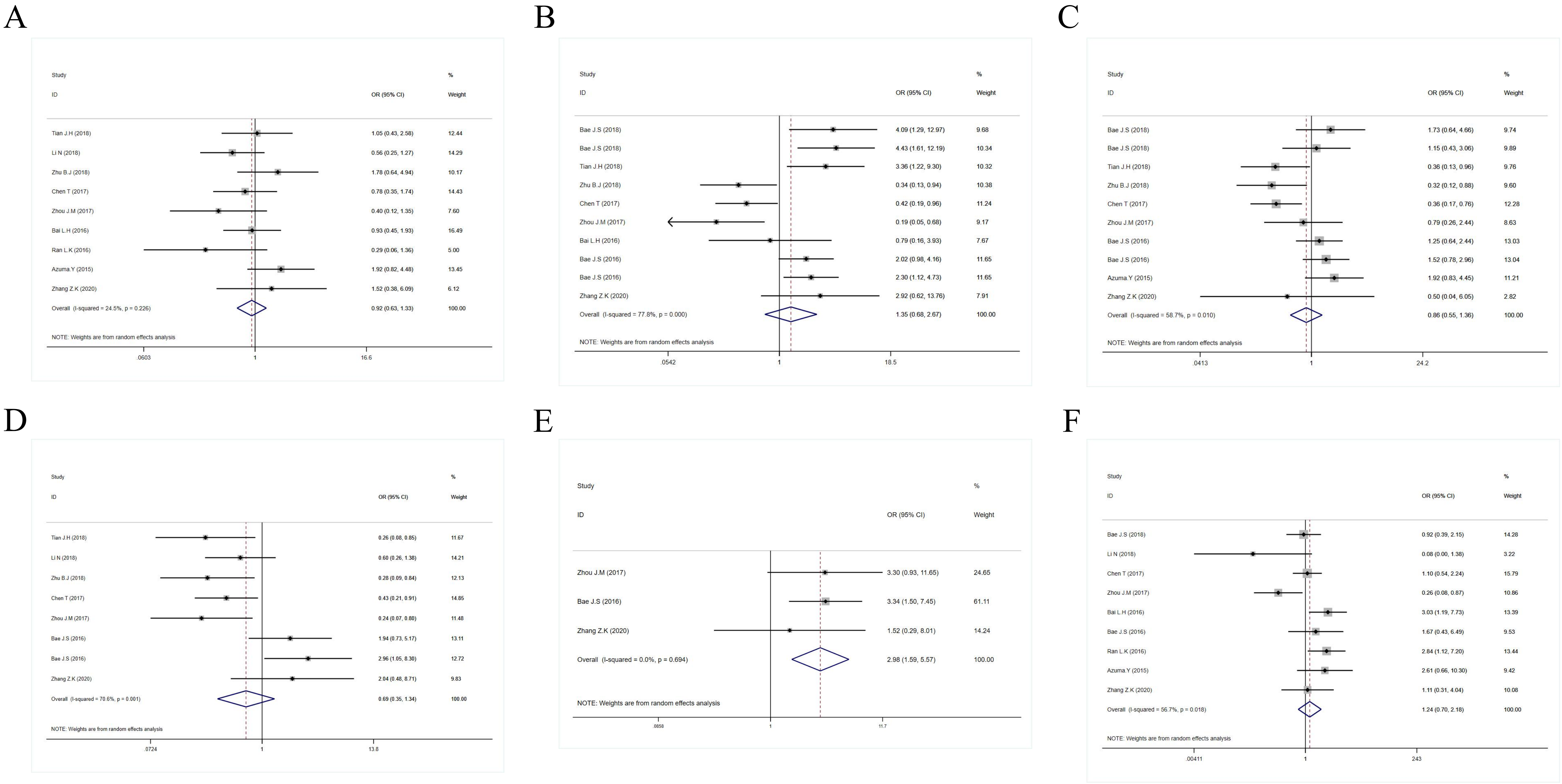

Figure S1 Forest plot for different clinicopathological parameters. (A) gender; (B) tumor differentiation; (C)Lymph node metastasis; (D) TNM stage; (E) diatant metastasis; (F)T staus
