# Supplementary material for: Clinicopathological and prognostic value of SIRT6 in patients with solid tumors: a meta-analysis and TCGA data review
Source: Cancer Cell Int. 2022 Feb 16;22:84. doi: 10.1186/s12935-022-02511-3 (PMC8848894; doi:10.1186/s12935-022-02511-3)
Supplement: Supplementary file 3 — Additional file 3: Figure S3. Forest plot for DFS of different clinicopathological parameters. [file 12935_2022_2511_MOESM3_ESM.pdf]

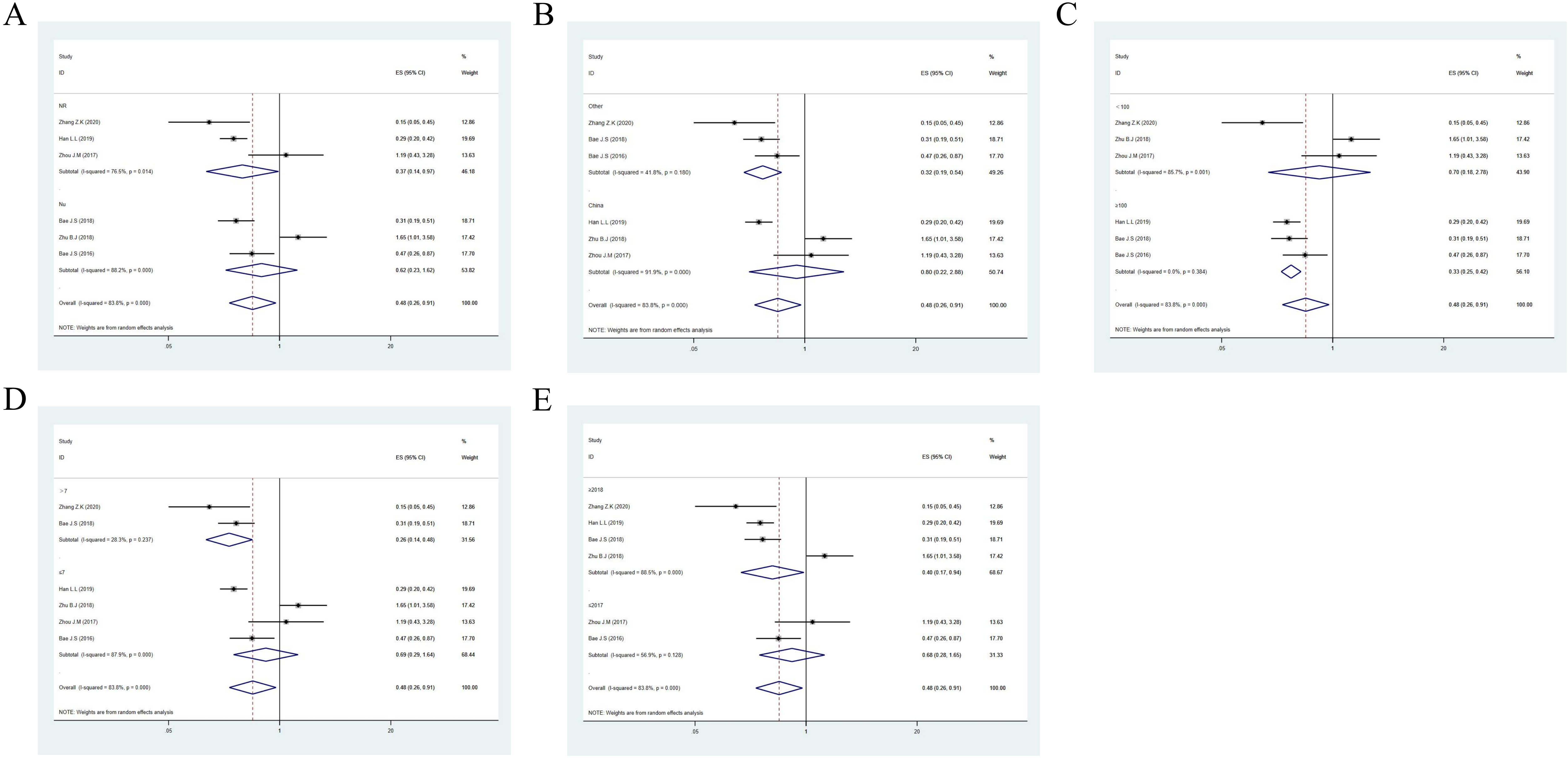

Figure S3 Forest plot for DFS of different clinicopathological parameters. (A) location; (B) region; (C)patients number; (D) NOS score; (E) publication year
