# Supplementary material for: Clinicopathological and prognostic value of SIRT6 in patients with solid tumors: a meta-analysis and TCGA data review
Source: Cancer Cell Int. 2022 Feb 16;22:84. doi: 10.1186/s12935-022-02511-3 (PMC8848894; doi:10.1186/s12935-022-02511-3)
Supplement: Supplementary file 4 — Additional file 4: Table S1. Summarized data of clinical and pathological parameters from the eligible studies. [file 12935_2022_2511_MOESM4_ESM.docx]

**Supplementary Table** Summarized data of clinical and pathological parameters from the eligible studies

| **Year** | **First author** | **Location** | **Gender** | | | | **Tumor differentiation** | | | | **T status** | | | | **Lymph node metastasis** | | | | **Distant metastasis** | | | | **TNM stage** | | | |
| --- | --- | --- | --- | --- | --- | --- | --- | --- | --- | --- | --- | --- | --- | --- | --- | --- | --- | --- | --- | --- | --- | --- | --- | --- | --- | --- |
|  |  |  | **Male** | | **Female** | | **Poor/Moderate/undifferentiated** | | **Well/**  **differentiated** | | **T3-4** | | **T1-2** | | **Yes** | | **No** | | **Yes** | | **No** | | **Ⅲ-Ⅳ** | | **Ⅰ-Ⅱ** | |
|  |  |  | **+** | **-** | **+** | **-** | **+** | **-** | **+** | **-** | **+** | **-** | **+** | **-** | **+** | **-** | **+** | **-** | **+** | **-** | **+** | **-** | **+** | **-** | **+** | **-** |
| 2020 | Zhang Z.K | NR | 13 | 12 | 5 | 7 | 15 | 12 | 3 | 7 | 9 | 9 | 9 | 10 | 1 | 2 | 17 | 17 | 4 | 3 | 14 | 16 | 14 | 12 | 4 | 7 |
| 2019 | Han L.L | NR | NR | NR | NR | NR | NR | NR | NR | NR | NR | NR | NR | NR | NR | NR | NR | NR | NR | NR | NR | NR | NR | NR | NR | NR |
| 2018 | Bae J.S | Nu | NR | NR | NR | NR | 32 | 45 | 4 | 23 | 12 | 24 | 24 | 44 | 9 | 11 | 27 | 57 | NR | NR | NR | NR | NR | NR | NR | NR |
| 2018 | Tian J.H | Nu | 13 | 28 | 15 | 34 | 11 | 10 | 17 | 52 | NR | NR | NR | NR | 7 | 30 | 21 | 32 | NR | NR | NR | NR | 4 | 24 | 24 | 38 |
| 2018 | Li N | Nu | 25 | 29 | 26 | 17 | NR | NR | NR | NR | 43 | 44 | 6 | 0 | NR | NR | NR | NR | NR | NR | NR | NR | 17 | 20 | 34 | 24 |
| 2018 | Zhu B.J | Nu | 15 | 35 | 7 | 29 | 8 | 40 | 14 | 24 | NR | NR | NR | NR | 8 | 41 | 14 | 23 | NR | NR | NR | NR | 5 | 33 | 17 | 31 |
| 2017 | Chen T | Cy | 35 | 54 | 15 | 18 | 12 | 34 | 30 | 36 | 32 | 35 | 25 | 30 | 20 | 42 | 34 | 26 | NR | NR | NR | NR | 25 | 49 | 26 | 22 |
| 2017 | Zhou J.M | NR | 10 | 42 | 6 | 10 | 4 | 33 | 12 | 19 | 5 | 33 | 11 | 19 | 8 | 29 | 8 | 23 | 6 | 8 | 10 | 44 | 5 | 34 | 11 | 18 |
| 2016 | Bai L.H | NR | 87 | 32 | 41 | 14 | 121 | 44 | 7 | 2 | 40 | 6 | 88 | 40 | NR | NR | NR | NR | NR | NR | NR | NR | NR | NR | NR | NR |
| 2016 | Bae J.S | Nu | NR | NR | NR | NR | 46 | 47 | 16 | 33 | 5 | 4 | 57 | 76 | 29 | 33 | 33 | 47 | 23 | 12 | 39 | 68 | 11 | 8 | 51 | 72 |
| 2016 | Ran L.K | NR | 55 | 32 | 12 | 2 | NR | NR | NR | NR | 55 | 21 | 12 | 13 | NR | NR | NR | NR | NR | NR | NR | NR | NR | NR | NR | NR |
| 2015 | Azuma.Y | Cy | 40 | 25 | 15 | 18 | NR | NR | NR | NR | 9 | 3 | 46 | 40 | 25 | 13 | 30 | 30 | NR | NR | NR | NR | NR | NR | NR | NR |

**Abbreviations:** NR, Not Report; Nu, nucleus; Cy, Cytoplasm.
